# Socioeconomic and Regional Disparities in Long-Term Mortality Among Intensive Care Unit Survivors in South Korea: Nationwide Cohort Study

**DOI:** 10.2196/89127

**Published:** 2026-08-31

**Authors:** Nayeon Choi, Jaehoon Oh, Kyung Hun Yoo, Juncheol Lee, Sang Hwan Lee

**Affiliations:** 1 Department of Emergency Medicine, College of Medicine, Hanyang University, 222 Wangsimni-ro, Seongdong-Gu, Seoul, Republic of Korea, 82 2-2290-9825, 82 2-2290-9280; 2 Hanyang Emergency Medicine AI & Data Science Lab (HEADS), Department of Emergency Medicine, College of Medicine, Hanyang University, Seongdong-Gu, Seoul, Republic of Korea; 3 Department of Emergency Medicine, Hanyang University Seoul Hospital, Seongdong-Gu, Seoul, Republic of Korea

**Keywords:** socioeconomic status, geographical regional factors, intensive care unit, long-term mortality, health disparities

## Abstract

**Background:**

Long-term outcomes of intensive care unit (ICU) survivors remain a major public health concern. Despite the existence of a universal health care system in South Korea, socioeconomic and regional disparities may affect postcritical illness survival.

**Objective:**

In this nationwide cohort study, we aimed to examine the associations of socioeconomic and regional factors with long-term mortality among ICU survivors.

**Methods:**

We conducted a retrospective population-based cohort study using data from the Korean National Health Insurance Service (NHIS). We included adult patients (≥20 y) admitted to ICUs between 2015 and 2019 who survived to hospital discharge. Patients admitted for injury-related or external causes were excluded. Postdischarge socioeconomic status (SES) indicators included insurance type (National Health Insurance [NHI] vs Medical Aid [MA]), NHIS eligibility status, and income level quartiles. Residential area was analyzed separately as a regional contextual factor. The primary outcome was all-cause mortality for up to 8 years after discharge, which was estimated using the Kaplan-Meier method. Adjusted hazard ratios (aHRs) and 95% CIs were estimated using multivariable time-dependent Cox proportional hazards models stratified by age (20‐39 y, 40‐64 y, and ≥65 y)

**Results:**

Among 871,366 adult ICU survivors, 9.6% (n=83,766) were MA beneficiaries. Lower SES—defined by MA coverage, nonemployee NHIS eligibility categories, and lower income level—was consistently associated with higher long-term mortality. Compared with NHI enrollees, MA recipients had an aHR of 1.63 (95% CI 1.60‐1.66) among middle-aged adults and 1.48 (95% CI 1.38‐1.60) among younger adults. Dependents and self-employed insured household members had 2-fold to 3-fold higher mortality risk than employee-insured beneficiaries. A stepwise income-mortality gradient was evident across all age groups. Residence in other regions was also associated with higher mortality (aHR 1.06‐1.11). Younger individuals were particularly vulnerable to SES-related disparities.

**Conclusions:**

Despite South Korea’s universal insurance system, socioeconomic and regional disparities in long-term mortality persist among ICU survivors. Ensuring equitable access to post-ICU care, income security, and targeted social support may help promote sustainable recovery.

## Introduction

With the growing global population, the demand for intensive care services continues to increase. While advancements in critical care have reduced short-term mortality rates among critically ill patients, long-term outcomes remain a major concern, especially for those discharged from intensive care units (ICUs) [1-3]. ICU survivors frequently experience physical, cognitive, and mental health deficits, resulting in diminished quality of life and increased long-term mortality [4]. To enhance care for ICU survivors and their families, practitioners and researchers must collaborate in both inpatient and outpatient environments [5,6]. However, socioeconomic status (SES) and age may affect access to post-ICU care, continuity of follow-up care, and long-term recovery [7-9].

Recent evidence has revealed that both SES-related factors, such as insurance type and income level, and regional factors may substantially influence long-term outcomes [7,8,10,11]. In South Korea, individuals receiving Medical Aid (MA), which is indicative of low-income and socially disadvantaged populations, have higher postdischarge mortality rates than do National Health Insurance (NHI) beneficiaries [7,12]. Additionally, regional disparities, reflected in urban morphology, health care access, and postacute care resources, may influence recovery pathways and long-term outcomes [11]. Social determinants such as unemployment, declining marriage rates, and social isolation, which are more common among individuals with low SES, have been associated with negative health outcomes and may impede recovery following treatment in ICUs [9,13]. Although awareness of these associations is increasing, there remains insufficient evidence, particularly in high-income Asian countries with different health care and welfare systems.

In this study, we aimed to investigate the associations of SES indicators (health insurance type, National Health Insurance Service [NHIS] eligibility status, and income level) and residential area, as a regional contextual factor, with long-term mortality among adult ICU survivors in South Korea. Since older adults are particularly susceptible and age markedly influences post-ICU prognosis, we examined whether these associations differed across age groups to enhance our understanding of age-related disparities in postdischarge outcomes.

## Methods

### Data Sources and Setting

This population-based cohort study used data from the NHIS database of South Korea. The NHIS operates as a government-administered, single-payer system under the National Health Insurance Act, which mandates universal participation. Consequently, the system covers nearly the entire Korean population [14]. The NHIS database contains comprehensive information on both inpatient and outpatient medical services, including demographic characteristics (eg, age, sex, residence, and smoking history), hospital visit details (department visited, visit dates, and patient status), diagnoses coded according to the *International Classification of Diseases, 10th Revision* (*ICD-10*) [15], prescriptions, medical procedures, surgeries, medical costs, and mortality. Socioeconomic variables such as income level and insurance type (NHI or MA) were also included, whereas cosmetic and nonevidence-based treatments were excluded [14]. Given its nationwide scope and data integrity, the NHIS database is a reliable source of data for large-scale population-based research.

### NHIS in South Korea

In South Korea, universal health coverage comprises 2 programs: the NHI and MA. The NHI, introduced in 1977 and expanded to full coverage by 1989, was unified into a single-payer model in 2000 and currently insures about 98% of the population. Similar to Medicaid in the United States, the MA program provides public assistance to low-income individuals and includes beneficiaries of the National Basic Livelihood Security System [16]. Under the NHI, insured individuals are classified as either employee-insured or self-employed. Employees, public officials, school staff, and their dependents are classified as employee-insured. In contrast, dependents are not separately recognized among the self-employed; the household head is responsible for paying the entire premium for all family members, including spouses, children, and parents. Premiums for employee-insured individuals are based on income and shared between employees and employers, whereas those for the self-employed are determined by household income, assets, vehicle ownership, age, and sex [17].

### Study Design and Population

This was a retrospective longitudinal cohort study designed to evaluate long-term mortality. We extracted data from patients admitted to ICUs between January 2015 and December 2019. ICU admission was determined by the attending physicians, and patients were identified using health insurance claim codes AJ100 and AJ390. Only adults aged 20 years or older were included in the study. ICU survivors were defined as patients discharged from the hospital. Patients admitted for injury-related or external causes (eg, burns, trauma, poisoning, asphyxia, and anaphylaxis) were excluded using *ICD-10* codes beginning with “S” or “T.” For patients with multiple ICU admissions during the study period, only the first admission was analyzed. The ICU admission date was defined as the cohort entry date, and the hospital discharge date of the first admission was defined as the index date. Demographic variables (sex, age, smoking status, comorbidities, interventions, hospitalization type, and residential area) and socioeconomic variables (health insurance type, NHIS eligibility status, and income level) were obtained from the NHIS database. We analyzed the associations of postdischarge socioeconomic indicators and residential area with long-term mortality, stratified by age group (20‐39 y, 40‐64 y, and ≥65 y). Multimedia Appendix 1 depicts the data selection process, exclusion criteria, enrollment period, and follow-up duration.

### Socioeconomic Indicators and Regional Factors

Individual-level SES is commonly assessed using indicators such as income, educational attainment, occupation, and insurance-related measures [18]. SES was assessed using the following three individual-level indicators: (1) insurance type, (2) NHIS eligibility status, and (3) income level. Residential area was analyzed as an independent regional factor.

Substantial disparities have been reported in these SES indicators between beneficiaries of the MA and NHI programs. For example, the proportions of individuals with low income (95% vs 34%), those with education levels at or below the elementary school level (61% vs 40%), and those not participating in economic activity (82% vs 57%) differed markedly between MA and NHI recipients, respectively [19]. Medicaid eligibility in the United States has been validated as a reliable proxy for individual-level SES, and insurance type has therefore been widely used as a surrogate measure of SES [12,18-20]. Given the substantial socioeconomic differences between MA and NHI beneficiaries in South Korea, we used MA status as an operational indicator of lower SES.

NHIS eligibility status was used as an SES-related administrative indicator in this study. Because the NHIS database does not directly capture employment status, unemployment, occupation, or labor force participation, this variable was interpreted as an administrative health insurance eligibility category rather than as a direct measure of employment or unemployment. Based on administrative eligibility data, NHIS eligibility status was categorized as employee-insured beneficiaries, dependents of employee-insured beneficiaries, self-employed insured householders, self-employed insured household members, and MA beneficiaries. Although employment and labor market participation are closely related to SES in the prior literature [21], this “NHIS eligibility status” variable should not be interpreted as a direct measure of employment. Employed-insured beneficiaries are generally workplace enrollees in the Korean health insurance system, whereas self-employed insured householders are responsible for paying insurance premiums based on income and assets. Dependents of employee-insured beneficiaries and self-employed insured household members may include individuals with limited income or without independent premium-paying status. MA beneficiaries represent a socioeconomically vulnerable group, and previous data have shown that 82% do not participate in economic activity [19]. However, individuals outside the employee-insured category may still derive income from self-employment, pensions, property, and other sources. Therefore, the NHIS eligibility status variable was used as an SES-related administrative indicator rather than as a direct classification of individuals as employed or unemployed [16,17].

The income level was inferred from health insurance premiums. MA beneficiaries are exempt from paying premiums, whereas NHI subscribers are categorized into quartiles according to their premium levels. MA eligibility requires a household income within 40% of the national median household income [17]. Accordingly, the lowest-income category in this study comprised MA beneficiaries and NHI enrollees within the lowest premium quartile, representing the lowest socioeconomic stratum.

The residential area was analyzed as a regional contextual factor rather than as a proxy for individual-level SES. Regional disparities may affect health outcomes through mechanisms distinct from those associated with socioeconomic disadvantage. Residential regions were classified into the Seoul metropolitan area (Seoul, Incheon, and Gyeonggi Province), noncapital metropolitan cities (excluding the Seoul metropolitan area), and other regions.

### Outcomes

The primary outcome was long-term mortality over an 8-year follow-up period (until 2022). Mortality rates were stratified by SES indicators, including insurance type, NHIS eligibility status, income level, and residential area. Long-term mortality according to SES was evaluated by classifying ICU survivors into 3 age groups: 20 to 39 years, 40 to 64 years, and 65 years and older. Each age group was separately evaluated. The follow-up period began on the index date and ended on December 31, 2022, or the date of death, whichever occurred first. Mortality rates were assessed at 1, 2, 4, 6, and 8 years, as well as overall.

Furthermore, we evaluated long-term mortality by examining residential area in conjunction with other socioeconomic factors (insurance, NHIS eligibility status, and income level). For the additional descriptive analysis, the low-income group was defined as comprising MA beneficiaries and NHI enrollees in the lowest premium quartile. The characteristics of the deceased and survivors in the low-income group were compared.

### Statistical Analysis

Baseline characteristics of the study population are presented as mean (SD) for continuous variables and as frequencies (%) for categorical variables. Differences among age groups were assessed using ANOVA for continuous variables and the chi-square test for categorical variables. The 8-year cumulative mortality was estimated using the Kaplan-Meier method. Analyses were stratified by age group, individual SES indicators, and residential area. Group differences were assessed using log-rank tests. The absolute mortality risk (AMR) difference across socioeconomic indicators and residential area categories was calculated as the difference in the 8-year cumulative mortality rates (eg, MA vs NHI, quartile 1 vs quartile 4, or dependents vs employee-insured beneficiaries). AMR is expressed in percentage points (%), providing an intuitive measure of absolute inequality in long-term mortality. Because socioeconomic factors and residential area vary over time, time-dependent Cox proportional hazards regression models were constructed to evaluate their independent associations with long-term mortality. Residential area was also included as an independent regional factor.

To avoid multicollinearity, in the time-dependent Cox models that included covariates, each SES indicator (insurance type, NHIS eligibility status, and income level) was included in a separate model along with residential area. Adjusted hazard ratios (aHRs) with 95% CIs were reported after controlling for age, sex, smoking status, the Charlson Comorbidity Index (CCI) score, and intervention status (mechanical ventilation, renal replacement, extracorporeal membrane oxygenation, and vasopressor or inotropic drugs), indicating disease severity.

Because residential areas may reflect contextual disparities that interact with, but are not fully captured by, individual-level SES indicators, we conducted an additional analysis to examine whether the association between residential area and long-term mortality persisted after adjusting for insurance type, NHIS eligibility status, or income level. In addition, to better understand mortality patterns among the most socioeconomically vulnerable groups, we compared the clinical and sociodemographic characteristics of survivors and decedents among MA recipients and individuals in the lowest-income quartile using independent 2-tailed *t* tests or chi-square tests. Because the Korean NHIS database is a nationwide administrative claims database covering virtually the entire Korean population, the present analysis was not based on a complex survey sample. Therefore, survey-sampling weights were not applied. The exclusions shown in the flow diagram were applied to define the prespecified target population of adult ICU survivors with noninjury or nonexternal-cause admissions and complete essential covariate information, rather than to construct a sampled subset of the source population. Data analyses were performed using SAS Enterprise Guide (version 7.1; SAS Institute Inc) and R (version 4.0.3; R Foundation for Statistical Computing). All statistical tests were 2-sided, and significance was set at *P*<.05.

### Ethical Considerations

The study design was approved by the Institutional Review Board of Hanyang University Hospital (HYUH 2023-04-049) and the Health Insurance Review and Assessment Service (NHIS-2024-1-315). Informed consent was waived as data analyses were performed retrospectively using deidentified data generated by an independent technician at the NHIS facility who had no association with this project.

## Results

### Study Population

**Figure 1.**
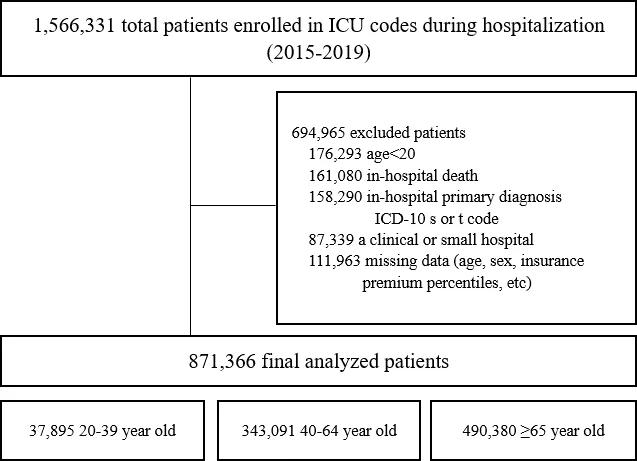
Flow diagram of participant selection. ICU: intensive care unit; *ICD: International Classification of Diseases*.

Using the NHIS database, we identified 1,566,331 adult patients admitted to ICUs between January 1, 2015, and December 31, 2019. After excluding patients aged younger than 20 years of age; those who died during hospitalization; individuals with injury-related or external causes (*ICD-10* codes beginning with “S” or “T”); and 111,963 patients lacking complete data on age, sex, insurance, or premium percentile, a total of 871,366 patients were included in the final analysis (Figure 1).

Table 1 summarizes the baseline characteristics of patients. The mean age was 66.0 (SD 14.4) years, and among 871,366 patients, 56.3% (n=490,380) were aged 65 years or older. Male patients accounted for 58.1% (n=506,307) of the cohort, with a more pronounced male sex predominance observed among younger adults. Most patients were covered by the NHI (n=787,600, 90.4%), while 9.6% (n=83,766) received MA. The mean CCI was 3.1 (SD 2.5), increasing significantly with age (*P*<.001). The proportion of MA beneficiaries increased with age (n=2460, 6.5% in the 20-year to 39-year group vs n=49,531, 10.1% in those ≥65 y). Regarding residence, 45.9% (n=399,804) and 36.8% (n=320,536) lived in the Seoul metropolitan area and other regions, respectively. The most frequent comorbidities were hypertension (n=539,389, 61.9%), dyslipidemia (n=537,561, 61.7%), and diabetes (n=375,003, 43.0%). Major ICU interventions included mechanical ventilation (n=167,287, 19.2%), use of vasopressors or inotropes (n=383,293, 43.9%), and renal replacement therapy (n=24,132, 2.8%). Notably, the overall mortality increased markedly with age (*P*<.001). The 1-year, 4-year, and 8-year mortality rates were 15.7% (n=136,379), 30.9% (n=269,382), and 39.6% (n=344,722), respectively. Long-term mortality was significantly higher among older adults (*P*<.001) than among younger adults. The main diagnoses for ICU patients were classified by organ system. Most diagnoses were circulatory system disorders (I00-I99; 418,886 cases), neoplasms (C00-D48; 144,719 cases), and digestive system diseases (K00-K93; 79,171 cases; Multimedia Appendix 2).

**Table 1.** Baseline characteristics of patients.

Characteristics	Total	20‐39 y	40‐64 y	≥65 y	*P* value
Number of patients, n (%)	871,366 (100)	37,895 (4.3)	343,091 (39.4)	490,380 (56.3)	—^a^
Age (y), mean (SD)	66.0 (14.4)	32.2 (5.6)	54.9 (6.5)	76.4 (7.3)	<.001
Sex, n (%)					<.001
Male	506,307 (58.1)	22,605 (59.7)	228,938 (66.7)	254,764 (51.9)	
Female	365,059 (41.9)	15,290 (40.3)	114,153 (33.3)	235,616 (48.0)	
Insurance status, n (%)					<.001
NHI^b^	787,600 (90.4)	35,435 (93.5)	311,316 (90.7)	440,849 (89.9)	
MA^c^	83,766 (9.6)	2460 (6.5)	31,775 (9.3)	49,531 (10.1)	
NHIS^d^ eligibility status, n (%)					<.001
Employee-insured	158,133 (18.1)	15,858 (41.8)	109,621 (31.9)	32,654 (6.7)	
Dependents	360,738 (41.4)	8710 (22.9)	81,238 (23.7)	270,790 (55.2)	
Self-employed-insured	174,541 (20.0)	4707 (12.4)	83,409 (24.3)	86,425 (17.6)	
Household members	94,188 (10.8)	6160 (16.3)	37,048 (10.8)	50,980 (10.4)	
MA	83,766 (9.6)	2460 (6.5)	31,775 (9.3)	49,531 (10.1)	
Income level, n (%)					<.001
MA	83,766 (9.6)	2460 (6.5)	31,775 (9.3)	49,531 (10.1)	
Quartile 1 (lowest)	134,793 (15.5)	6287 (16.6)	61,059 (17.8)	67,447 (13.8)	
Quartile 2	217,131 (24.9)	13,918 (36.7)	101,252 (29.5)	101,961 (20.8)	
Quartile 3	217,937 (25.0)	10,269 (27.1)	83,674 (24.4)	123,994 (25.3)	
Quartile 4 (highest)	217,739 (24.9)	4961 (13.1)	65,331 (19.0)	147,447 (30.1)	
Residential area, n (%)					<.001
Seoul metropolitan area	399,804 (45.9)	19,840 (52.4)	165,356 (48.2)	214,608 (43.8)	
Noncapital metropolitan cities	151,026 (17.3)	6777 (17.9)	63,637 (18.5)	80,612 (16.4)	
Other regions	320,536 (36.8)	11,278 (29.8)	114,098 (33.3)	195,160 (39.8)	
Smoking status, n (%)					<.001
Nonsmoker	334,314 (38.4)	11,095 (29.3)	119,875 (34.9)	203,344 (41.5)	
Ex-smoker	157,722 (18.1)	4974 (13.1)	75,276 (21.9)	77,472 (15.8)	
Current smoker	101,868 (11.7)	5269 (13.9)	64,673 (18.9)	31,926 (6.5)	
Unknown	277,462 (31.8)	16,557 (43.7)	83,267 (24.3)	177,638 (36.2)	
Comorbidities, n (%)^e^					
CCI^f^ score, mean (SD)	3.1 (2.5)	1.4 (1.7)	2.5 (2.3)	3.6 (2.5)	<.001
Hypertension	539,389 (61.9)	7037 (18.6)	162,449 (47.3)	369,903 (75.4)	<.001
Dyslipidemia	537,561 (61.7)	11,877 (31.3)	189,637 (55.3)	336,047 (68.5)	<.001
CAD^g^	42,013 (4.8)	367 (1.0)	12,008 (3.5)	29,638 (6.0)	<.001
Diabetes	375,003 (43.0)	5972 (15.8)	119,906 (34.9)	249,125 (50.8)	<.001
Stroke	117,265 (13.5)	1019 (2.7)	24,652 (7.2)	91,594 (18.7)	<.001
Acute myocardial infarction	36,369 (4.2)	296 (0.8)	10,470 (3.1)	25,603 (5.2)	<.001
Congestive heart failure	118,867 (13.6)	1717 (4.5)	28,357 (8.3)	88,793 (18.1)	<.001
Peripheral vascular disease	169,758 (19.5)	1715 (4.5)	43,781 (12.8)	124,262 (25.3)	<.001
Cerebral vascular accident	225,806 (25.9)	2734 (7.2)	58,707 (17.1)	164,365 (33.5)	<.001
Dementia	92,191 (10.6)	120 (0.3)	5202 (1.5)	86,869 (17.7)	<.001
Pulmonary disease	412,710 (47.4)	12,264 (32.4)	127,131 (37.1)	273,315 (55.7)	<.001
Connective tissue disorder	47,606 (5.5)	1062 (2.8)	14,924 (4.3)	31,620 (6.4)	<.001
Peptic ulcer	337,030 (38.7)	8922 (23.5)	117,574 (34.3)	210,534 (42.9)	<.001
Liver disease	74,420 (8.5)	2117 (5.6)	35,470 (10.3)	36,833 (7.5)	<.001
Diabetes complications	111,407 (12.8)	1465 (3.9)	34,279 (9.9)	75,663 (15.4)	<.001
Paraplegia	18,731 (2.1)	379 (1.0)	5099 (1.5)	13,253 (2.7)	<.001
Renal disease	76,937 (8.8)	1863 (4.9)	22,671 (6.6)	52,403 (10.7)	<.001
Cancer	156,399 (17.9)	3729 (9.8)	59,471 (17.3)	93,199 (19.0)	<.001
Metastatic cancer	24,803 (2.8)	800 (2.1)	11,312 (3.3)	12,691 (2.6)	<.001
Severe liver disease	10,418 (1.2)	331 (0.9)	5695 (1.7)	4392 (0.9)	<.001
HIV	428 (0.0)	52 (0.1)	249 (0.1)	127 (0.0)	<.001
Intervention, n (%)^e^					
Mechanical ventilation	167,287 (19.2)	9690 (25.6)	69,610 (20.2)	87,987 (17.9)	<.001
Renal replacement	24,132 (2.8)	1247 (3.3)	9240 (2.7)	13,645 (2.8)	<.001
Vasopressor or inotropic drugs	383,293 (43.9)	17,452 (46.1)	155,457 (45.3)	210,384 (42.9)	<.001
ECMO^h^	3861 (0.4)	448 (1.2)	2220 (0.6)	1193 (0.2)	<.001
Hospitalization type, n (%)					<.001
Tertiary hospital^i^	370,599 (42.5)	19,463 (51.4)	162,182 (47.3)	188,954 (38.5)	
General hospital	500,767 (57.5)	18,432 (48.6)	180,909 (52.7)	301,426 (61.5)	
Year of ICU^j^ admission, n (%)					<.001
2015	188,583 (21.6)	7715 (20.4)	73,418 (21.4)	107,450 (21.9)	
2016	180,033 (20.7)	7651 (20.2)	71,249 (20.8)	101,133 (20.6)	
2017	173,897 (19.9)	7514 (19.8)	68,636 (20.0)	97,747 (19.9)	
2018	163,623 (18.8)	7320 (19.3)	64,601 (18.8)	91,702 (18.7)	
2019	165,230 (18.9)	7695 (20.3)	65,187 (18.9)	92,348 (18.8)	
Long-term death (y), n (%)^k^					
1	136,379 (15.7)	2238 (5.9)	28,351 (8.3)	105,790 (21.6)	<.001
2	190,367 (21.8)	3184 (8.4)	41,640 (12.1)	145,543 (29.7)	<.001
4	269,382 (30.9)	4419 (11.7)	59,839 (17.4)	205,124 (41.8)	<.001
6	321,146 (36.8)	5129 (13.5)	71,693 (20.9)	244,324 (49.8)	<.001
8	344,722 (39.6)	5434 (14.3)	76,963 (22.4)	262,325 (53.5)	<.001

a Not applicable.

b NHI: National Health Insurance.

c MA: Medical Aid.

d NHIS: National Health Insurance Service.

e Comorbidities and interventions are not mutually exclusive; n and % reflect patients with each condition or intervention and do not sum to N or 100%.

f CCI: Charlson Comorbidity Index.

g CAD: coronary artery disease.

h ECMO: extracorporeal membrane oxygenation.

i Ministry-designated tertiary hospitals in Korea.

j ICU: intensive care unit.

k Long-term death values are cumulative across time points; n and % overlap across subcategories and do not sum to N or 100%.

### SES and Regional Disparities in Long-Term Mortality

**Figure 2.**
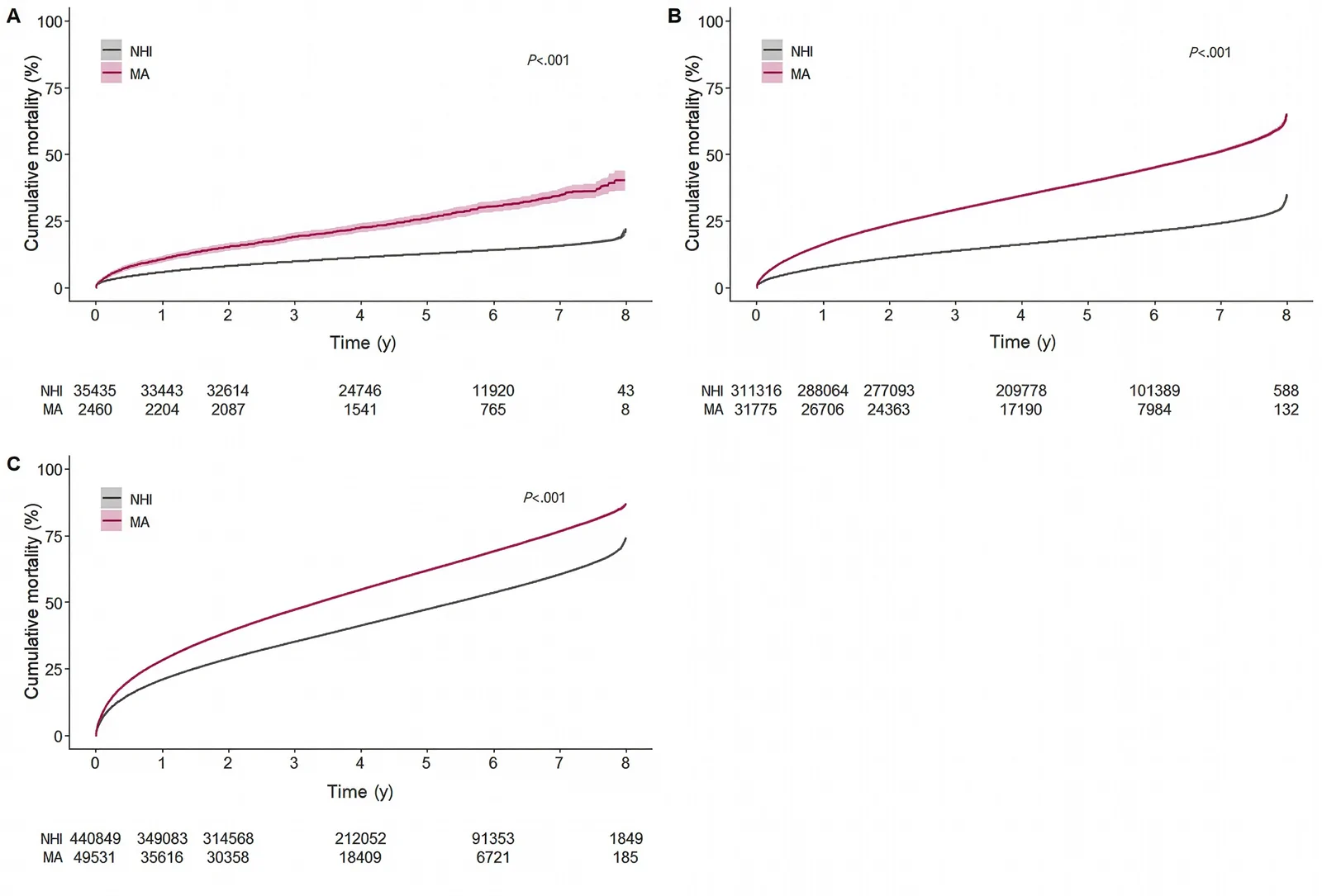
Cumulative mortality curves by insurance status, stratified by age group: (A) 20 to 39 years; (B) 40 to 64 years; and (C) 65 years and older. NHI: National Health Insurance; MA: Medical Aid.

**Figure 3.**
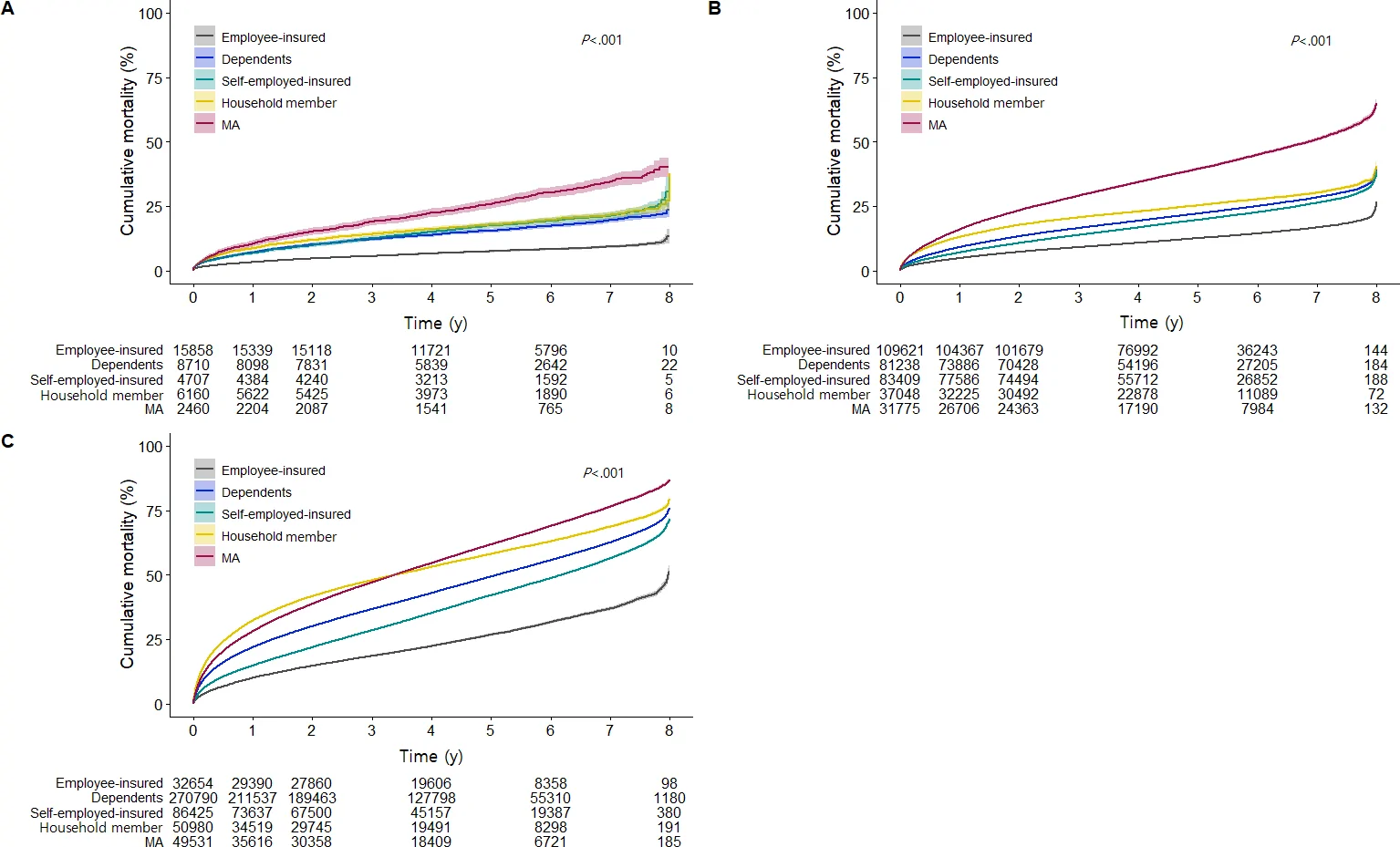
Cumulative mortality curves by National Health Insurance Service (NHIS) eligibility status, stratified by age group: (A) 20 to 39 years; (B) 40 to 64 years; and (C) 65 years and older. MA: Medical Aid.

**Figure 4.**
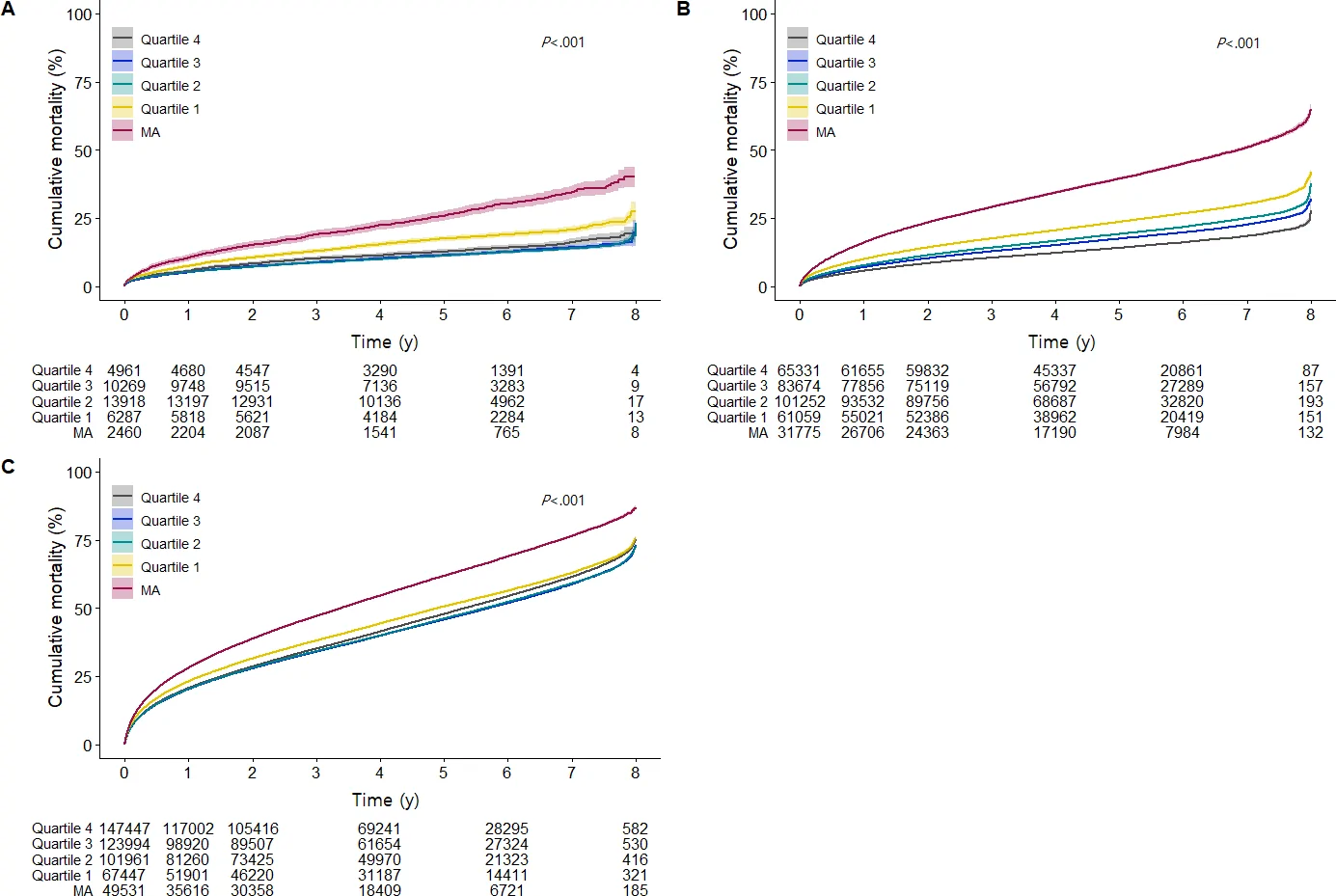
Cumulative mortality curves by income level, stratified by age group: (A) 20 to 39 years; (B) 40 to 64 years; and (C) 65 years and older. MA: Medical Aid.

**Figure 5.**
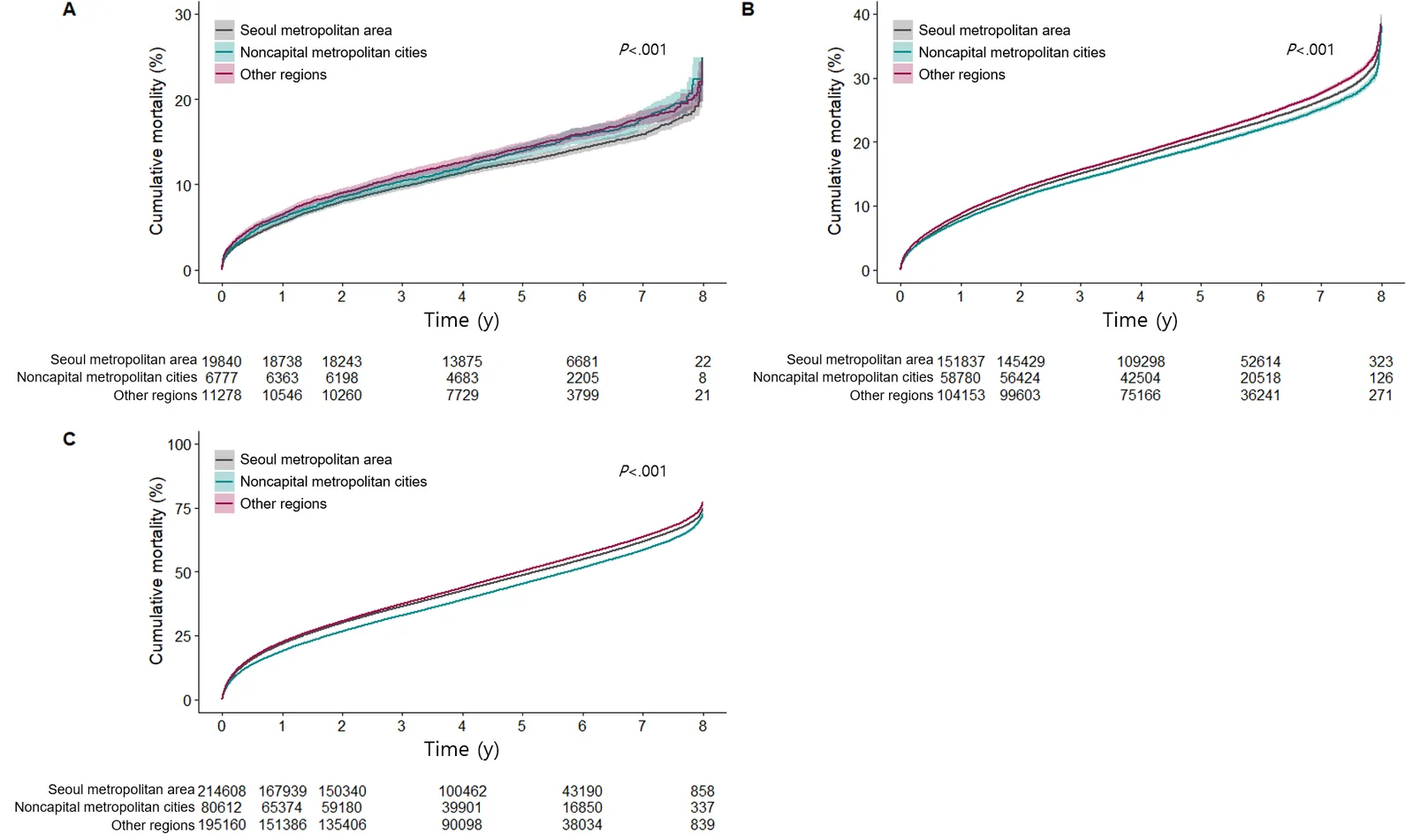
Cumulative mortality curves by residential area, stratified by age group: (A) 20 to 39 years; (B) 40 to 64 years; and (C) 65 years and older.

In all age groups, the cumulative mortality curves differed significantly according to SES indicators (Figures 2-5; log-rank test, *P*<.001). Specifically, MA beneficiaries had higher mortality than did NHI beneficiaries (Figure 2); dependents and self-employed household members had poorer survival than did employee-insured individuals (Figure 3); and mortality increased stepwise with decreasing income, with MA beneficiaries exhibiting the worst outcomes, even when compared with those in the lowest-income quartile (Figure 4). Patients residing in other regions also exhibited higher mortality rates than those residing in the Seoul metropolitan area or noncapital metropolitan cities (Figure 5). The 8-year cumulative mortality (%) and AMR differences by SES indicators and age groups are presented in Multimedia Appendix 3. The 8-year AMR differences between MA and NHI were 29.8% in middle-aged adults and 17.9% in young adults. Across all socioeconomic indicators, the absolute mortality gap increased as SES decreased, with the steepest gradients observed for insurance type, NHIS eligibility status, and income level, particularly among middle-aged adults (40‐64 y old).

Conversely, regional disparities were relatively small, with absolute differences of less than 3% across age categories, although differences by residential area remained statistically significant.

### Multivariable Analysis of the Association Between SES, Residential Area, and Long-Term Mortality

#### Socioeconomic Indicators and Residential Area

**Figure 6.**
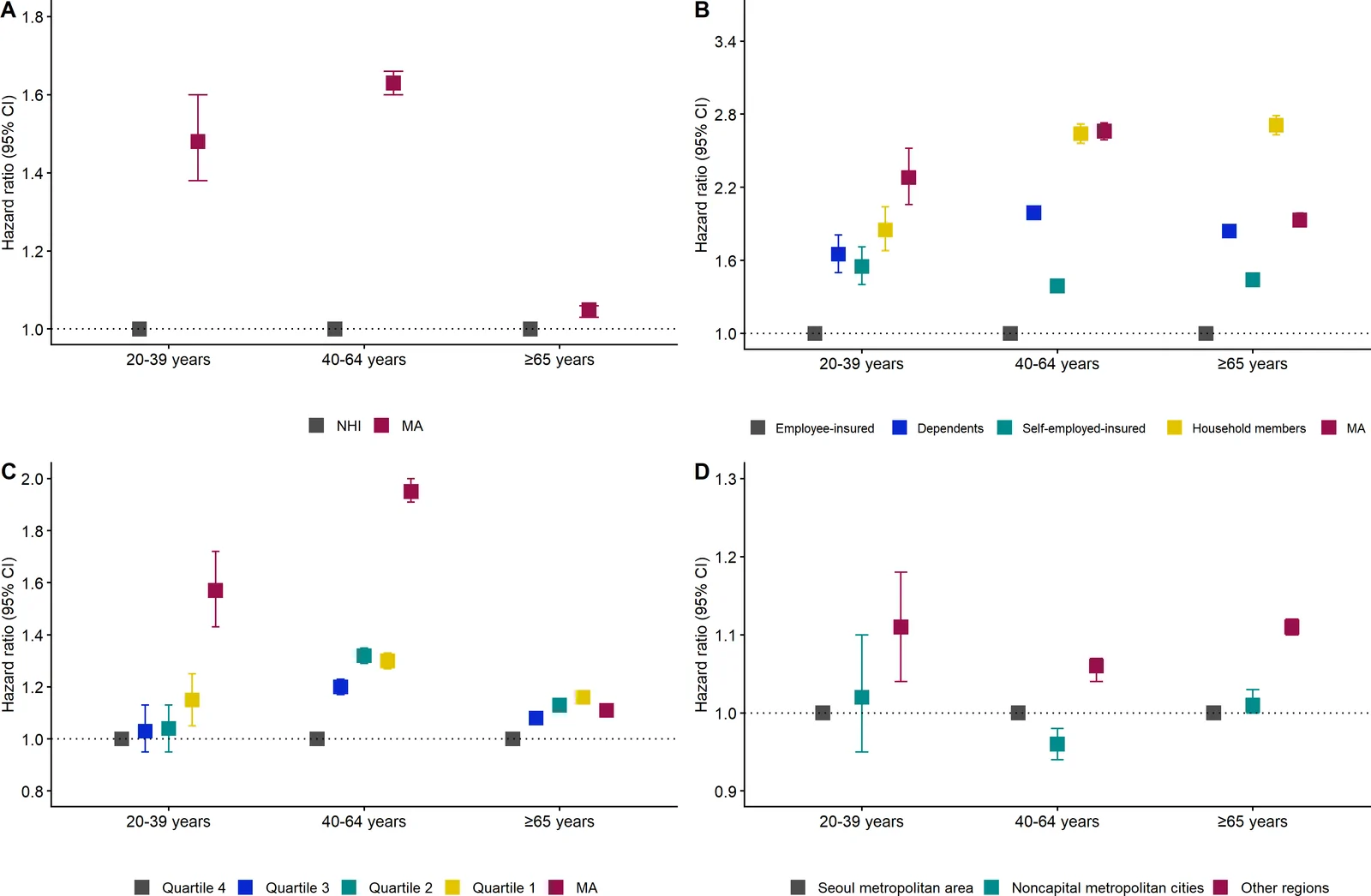
Forest plots of adjusted hazard ratios for mortality according to socioeconomic factors and residential area, stratified by age group: (A) insurance status; (B) National Health Insurance Service (NHIS) eligibility status; (C) income level; and (D) residential area. Adjusted for age, sex, smoking status, the Charlson Comorbidity Index, and interventions. NHI: National Health Insurance; MA: Medical Aid.

Figure 6 presents forest plots of the aHRs for mortality according to socioeconomic indicators and residential area stratified by age group. Multimedia Appendix 4 summarizes the time-dependent Cox regression analyses evaluating the associations of socioeconomic indicators and residential area with long-term mortality among ICU survivors stratified by age. After adjusting for age, sex, smoking status, CCI score, and intervention status, both socioeconomic indicators and residential area were independently associated with long-term postdischarge mortality.

#### Insurance Status

In patients aged 65 years or older, the risk of mortality after full adjustment was marginally higher among MA beneficiaries than among NHI enrollees (8-y aHR 1.05, 95% CI 1.03‐1.06). In contrast, younger age groups exhibited progressively higher mortality risk associated with MA coverage. Among individuals aged 40 to 64 years, the aHR increased from 1.28 (95% CI 1.24‐1.32) at 1 year to 1.63 (95% CI 1.60‐1.66) at 8 years. Among those aged 20 to 39 years, the aHR increased from 1.12 (95% CI 0.98‐1.28) at 1 year to 1.48 (95% CI 1.38‐1.60) at 8 years.

#### NHIS Eligibility Status

NHIS eligibility categories were associated with mortality. Compared with employee-insured beneficiaries, dependents of employee-insured beneficiaries, self-employed-insured householders, and self-employed-insured household members had significantly higher long-term mortality in all age groups. Among individuals aged 40 to 64 years, the 8-year aHRs were 1.99 (95% CI 1.94‐2.04) for dependents of employee-insured beneficiaries and 2.64 (95% CI 2.56‐2.72) for self-employed-insured household members. Among those aged 65 years or older, 8-year aHRs reached 1.84 (95% CI 1.80‐1.89) for dependents of employee-insured beneficiaries and 2.71 (95% CI 2.63‐2.79) for self-employed-insured household members.

#### Income Level

A distinct income-mortality gradient was observed across all age groups. When MA beneficiaries were analyzed along with income quartiles, their mortality risks were comparable to or exceeded those of the lowest-income quartile. Compared with the highest-income group, the 8-year mortality hazard increased progressively from the fourth quartile to the lowest-income group and to MA beneficiaries:

1. 20 to 39 years: 8-year aHR for the first quartile 1.15 (95% CI 1.05-1.25) and MA 1.57 (95% CI 1.43-1.72)
2. 40 to 64 years: 8-year aHR for the first quartile 1.30 (95% CI 1.27-1.33) and MA 1.95 (95% CI 1.91-2.00)
3. 65 years and older: 8-year aHR for the first quartile 1.16 (95% CI 1.14-1.17) and MA 1.11 (95% CI 1.10-1.13)

#### Residential Area

Compared with residents of the Seoul metropolitan area, those living in other regions consistently had higher mortality risks (20‐39 y: 8-y aHR 1.11, 95% CI 1.04‐1.18; 40‐64 y: 8-y aHR 1.06, 95% CI 1.04‐1.07; and ≥65 y: 8-y aHR 1.11, 95% CI 1.10‐1.12). This difference remained significant even after adjusting for individual-level socioeconomic indicators (Multimedia Appendix 5).

### Characteristics of the Survivors and Decedents Within the Low-Income Group (the MA and First Quartile Groups)

Among patients with low SES (n=218,559), the mean age was 65.9 (SD 14.8) years, and 55.3% (n=120,863) were male patients. Compared with survivors (mean 60.9, SD 13.5 y), decedents were significantly older (mean 71.4, SD 14.2 y; *P*<.001) and had a higher comorbidity burden (mean CCI 3.9, SD 2.7 vs mean 2.7, SD 2.3; *P*<.001). The proportion of MA beneficiaries was greater among decedents (48,050/103,190, 46.6% vs 35,716/115,369, 30.9%; *P*<.001), and the proportion of residents in other regions was higher among decedents than among survivors (41,713/103,190, 40.4% vs 41,860/115,369, 36.3%; *P*<.001; Multimedia Appendix 6).

## Discussion

### Age-Related Differences in Socioeconomic Disparities

This nationwide cohort study demonstrated substantial socioeconomic and regional disparities in long-term mortality among ICU survivors in South Korea. Using time-dependent Cox models that accounted for temporal changes in SES and residential area, and that adjusted for clinical severity, we found that lower SES, defined by MA coverage, nonemployee NHIS eligibility categories, and low income, was independently associated with higher postdischarge mortality. In addition, residence in other regions was associated with an increased mortality risk.

The Kaplan-Meier survival curves clearly demonstrated these differences across all SES indicators and residential area categories, and the log-rank tests (all *P*’s<.001) confirmed that the curves differed significantly. This disparity was most evident among younger and middle-aged adults, with cumulative mortality rising more sharply in socioeconomically disadvantaged groups. One notable finding of this study was that socioeconomic gradients were most pronounced among younger adults, suggesting that SES-related factors may exert a greater proportional influence among populations with a lower baseline mortality risk. This pattern aligns with the “relative deprivation hypothesis,” which posits that socioeconomic disadvantage exerts a more pronounced proportional impact on mortality among populations characterized by a low baseline risk [22]. In particular, younger MA beneficiaries or individuals with low incomes may encounter greater obstacles to returning to work, social engagement, or psychological recovery, which are crucial for long-term health following critical illness [23]. Building on this, the more pronounced SES-related disparities observed among younger age groups, especially among MA beneficiaries, may reflect the heterogeneous nature of this population. Young MA recipients may include individuals with severe chronic conditions, disabilities, mental health disorders, or marked social vulnerability, all of which may contribute to poor long-term outcomes after ICU discharge. Therefore, the observed disparities may not solely reflect economic disadvantages but rather the cumulative effect of multiple medical and social risk factors within these subgroups.

### Socioeconomic Inequality and Long-Term Mortality After Critical Illness

In the United States, Medicaid users and uninsured patients are less likely to receive critical care services and are more likely to be withdrawn from life support, indicating that lack of insurance independently increases the risk of mortality [24]. ICU patients with public or no insurance had higher short-term and long-term mortality rates than did those with private insurance [25]. Similar evidence from Australia demonstrates that socioeconomic disadvantages negatively affect long-term survival after ICU admission [26]. Consistent with these findings, Cha et al [7] reported that the 1-year mortality was 1.31 times higher among MA recipients than among NHI enrollees in a propensity-matched cohort of 2495 ICU survivors in South Korea.

SES is well-established as a key determinant of cardiovascular health, particularly in acute coronary syndrome [27,28]. A 14-year Korean registry study involving 4873 cardiac arrest survivors revealed a statistically significant reduction in long-term survival rates among patients with MA (aHR 1.52) compared with NHI beneficiaries [12]. Nevertheless, a study conducted in Singapore revealed no significant correlation between SES and out-of-hospital cardiac arrest survival outcomes, likely attributable to the availability of affordable and equitable health care for all socioeconomic strata [29].

These studies corroborate our findings that socioeconomic factors influence survival, even within a universal health care framework. Specifically, compared with NHI enrollees, younger MA beneficiaries in our cohort exhibited up to a 1.5-fold increase in 8-year mortality risk, whereas the disparity was negligible among older adults (aHR 1.05, 95% CI 1.03‐1.06). The persistence of SES-related disparities in a system characterized by nearly universal insurance coverage highlights that nonfinancial barriers, such as social isolation, restricted access to rehabilitation, limited continuity of care, and insufficient postacute care, remain crucial factors influencing post-ICU recovery [30-32].

### NHIS Eligibility Status and Income as Markers of Post-ICU Vulnerability

In our study, differences across the NHIS eligibility categories were particularly evident. Compared with individuals with employee insurance, dependent and self-employed household members had a 2-fold to 3-fold increased mortality risk even after multivariable adjustment. A consistent income-mortality gradient was observed across all age groups, with mortality rates increasing from the highest to the lowest-income quartiles. MA beneficiaries, who were the most economically disadvantaged group, had an even higher risk of mortality than did those in the lowest-income NHI quartile, suggesting that MA status may capture broader socioeconomic and clinical vulnerabilities beyond income level alone. Our results indicate that clinical and sociodemographic characteristics vary considerably even within low-SES groups. This finding underscores the importance of developing more refined and tailored post-ICU discharge management strategies rather than applying a one-size-fits-all approach to socioeconomic risk.

Oh et al [33] analyzed a cohort of over 6000 adults from a tertiary hospital in South Korea and found that, compared with office workers, unemployed patients experienced markedly higher mortality at both 30 days and 1-year post-ICU admission (83%). In a substantial Taiwanese cohort representing the general population (not restricted to ICU patients), involuntary unemployment was found to be correlated with an increased mortality risk (aHR 1.99) [34]. A meta-analysis of 52 studies revealed that approximately 60% of previously employed ICU survivors had resumed work 1 year postdischarge, and 68% after 5 years. The low rate of return to work was attributed to preexisting comorbidities and the ongoing physical, cognitive, and psychological effects of the critical illness. Even among individuals who return to work, many encounter job transitions, diminished working hours, or subsequent unemployment [35]. In Canada, a national cohort study of adults with severe trauma, including ICU patients, revealed a substantial return-to-work gap and a 19% reduction in average wages 3 years postinjury, with an employment rate of 79.3% compared with 91.7% in matched controls [36].

Collectively, these findings demonstrate that differences in workplace-based health surveillance, social capital, and continuity of care may affect long-term outcomes after serious illness. Job security and stable income seem to facilitate follow-up care, medication adherence, and the maintenance of healthy behaviors during long-term recovery.

### Geographic Disparities in Access to Post-ICU Care

Patients living in other regions had modest but consistently higher mortality risks across all age groups (8-year aHR 1.06‐1.11). In an additional analysis, residence in other regions was consistently associated with higher long-term mortality than with residence in the Seoul metropolitan area across all age groups, even after adjustment for individual socioeconomic indicators, such as insurance type, NHIS eligibility status, and income level. These findings suggest that residential areas may capture regional contextual disparities in post-ICU outcomes that are not fully explained by individual-level socioeconomic disadvantages alone.

A population-based study comparing rural and urban residents hospitalized for COVID-19 found that rural residents had a 22% higher risk of long-term mortality than did their urban counterparts [11]. Likewise, a retrospective analysis of Medicare beneficiaries in the United States, analogous to the NHI in Korea, revealed a persistent rural-urban disparity in mortality after discharge from acute care hospitals [37].

Communities in other regions may experience multiple structural disadvantages, such as increased distances to tertiary hospitals, constrained ICU capacity, shortages of specialists, and limited access to advanced medical equipment. Consequently, patients in these areas often have poorer postdischarge survival rates. Moreover, elevated long-term mortality may indicate both economic disparities and nonfinancial obstacles, including insufficient post-ICU rehabilitation resources, the absence of family or caregiver support, nursing workforce shortages, inadequate compliance with follow-up appointments, and difficulties in remote health monitoring.

Although the Korean health care system offers universal insurance coverage, health care resources and postacute care infrastructure are predominantly located in urban areas. These results highlight the necessity for region-specific post-ICU follow-up programs and telemedicine-based continuity of care to mitigate geographic disparities in long-term outcomes after critical illness.

### Limitations

Some limitations of this study should be acknowledged. First, the NHIS database lacks detailed clinical information, such as physiological and laboratory variables (eg, Acute Physiology and Chronic Health Evaluation II, Sequential Organ Failure Assessment scores, or vital parameters). Although surrogate indicators (mechanical ventilation, renal replacement therapy, vasopressor use, and extracorporeal membrane oxygenation) were used to approximate illness severity, residual confounding owing to unmeasured clinical heterogeneity may persist. Second, SES indicators, including insurance type, NHIS eligibility status, and income level, were inferred from administrative and premium-based data rather than direct measures of education, occupation, household wealth, or actual labor force participation. While these proxies have been validated in prior population-based research, they may not fully capture multidimensional socioeconomic contexts, such as education or social capital. Third, behavioral and psychosocial variables (eg, marital status, mental health, family or social support, physical activity, lifestyle factors, and health literacy) were not included in the NHIS data. These unmeasured confounders may partly explain the observed SES-mortality association, particularly among younger and working-age adults. Additionally, variations in hospital resources and local post-ICU care infrastructure (eg, rehabilitation and nursing facilities) were not directly measured. Furthermore, although sampling weights were not applied because this study analyzed the complete eligible nationwide NHIS cohort rather than a sampled population, potential selection bias due to exclusions or missing data cannot be completely ruled out. Future studies could assess the impact of such bias using sensitivity analyses, including weighting methods or multiple imputation, where appropriate. Finally, owing to the retrospective observational design, causal interpretations should be made with caution, and unmeasured confounding may remain despite extensive covariate adjustment. Future studies integrating detailed clinical data, patient-reported outcomes, and regional health care resource mapping are required to elucidate the causal pathways linking socioeconomic and regional factors to long-term post-ICU mortality.

### Conclusions

In this national cohort of ICU survivors, disparities in individual SES and residential region were independently associated with long-term mortality, even within a universal health care system. The impact of low SES was particularly strong among younger and middle-aged individuals. For these groups, low income and vulnerable NHIS eligibility categories were more strongly associated with long-term mortality, even after accounting for age and comorbidities. Residence in other regions was consistently associated with higher long-term mortality across all age groups. These findings suggest that socioeconomic disadvantage and regional disparities influence survival trajectories beyond acute illnesses, highlighting the persistent social gradient in health outcomes. Future policies should prioritize post-ICU transitional care networks, community-based rehabilitation, and risk screening during discharge planning to address socioeconomic and regional vulnerabilities.

## Supplementary material

10.2196/89127 Multimedia Appendix 1 Study design and assessment windows.

10.2196/89127 Multimedia Appendix 2 Primary diagnoses at intensive care unit admission classified by system.

10.2196/89127 Multimedia Appendix 3 Eight-year cumulative mortality (%) and difference in absolute mortality risk by socioeconomic indicators, residential area, and age groups.

10.2196/89127 Multimedia Appendix 4 Adjusted associations between age (y), socioeconomic factors, residential area, and long-term mortality among intensive care unit survivors.

10.2196/89127 Multimedia Appendix 5 Age group-specific associations between residential area and long-term mortality among intensive care unit survivors after adjustment for individual socioeconomic indicators.

10.2196/89127 Multimedia Appendix 6 Comparison of characteristics between survivors and decedents in the low-income group (Medical Aid and first quartile).

## Abbreviations

aHR: adjusted hazard ratio
AMR: absolute mortality risk
CCI: Charlson Comorbidity Index
HYUH: Hanyang University Hospital
*ICD-10*: *International Classification of Diseases, 10th Revision*
ICU: intensive care unit
MA: Medical Aid
NHI: National Health Insurance
NHIS: National Health Insurance Service
SES: socioeconomic status
