# Supplementary material for: Socioeconomic and Regional Disparities in Long-Term Mortality Among Intensive Care Unit Survivors in South Korea: Nationwide Cohort Study
Source: JMIR Public Health Surveill. 2026 Aug 31;12:e89127. doi: 10.2196/89127 (PMC13529258; doi:10.2196/89127)
Supplement: Supplementary file 2 [file publichealth-v12-e89127-s002.docx]

**Supplementary Table 1**. Primary diagnoses at ICU admission classified by system

| **ICD-10** | **N** |
| --- | --- |
| Diseases of the circulatory system (I00-I99) | 418,886 |
| Neoplasms (C00-D48) | 144,719 |
| Diseases of the digestive system (K00-K93) | 79,171 |
| Diseases of the respiratory system (J00-J99) | 63,304 |
| Diseases of the genitourinary system (N00-N99) | 37,574 |
| Diseases of the musculoskeletal system and connective tissue (M00-M99) | 25,962 |
| Diseases of the nervous system (G00-G99) | 23,801 |
| Certain infectious and parasitic diseases (A00-B99) | 22,156 |
| Endocrine, nutritional and metabolic diseases (E00-E90) | 17,766 |
| Symptoms, signs and abnormal clinical and laboratory findings, NEC (R00-R99) | 16,235 |
| Mental and behavioral disorders (F00-F99) | 6,261 |
| Factors influencing health status and contact with health services (Z00-Z99) | 5,122 |
| Congenital malformations, deformations and chromosomal abnormalities (Q00-Q99) | 4,861 |
| Diseases of the blood and blood-forming organs and certain disorders involving the immune mechanism (D50-D89) | 2,022 |
| Diseases of the skin and subcutaneous tissue (L00-L99) | 1,596 |
| Pregnancy, childbirth and the puerperium (O00-O99) | 1,304 |
| Diseases of the ear and mastoid process (H60-H95) | 384 |
| Diseases of the eye and adnexa (H00-H59) | 174 |
| Codes for special purposes (U00-U99) | 36 |
| Certain conditions originating in the perinatal period (P00-P96) | 32 |

ICD, International Classification of Diseases; ICU, intensive care unit.
