# Supplementary material for: Socioeconomic and Regional Disparities in Long-Term Mortality Among Intensive Care Unit Survivors in South Korea: Nationwide Cohort Study
Source: JMIR Public Health Surveill. 2026 Aug 31;12:e89127. doi: 10.2196/89127 (PMC13529258; doi:10.2196/89127)
Supplement: Supplementary file 3 [file publichealth-v12-e89127-s003.docx]

**Supplementary Table 2**. Eight-year cumulative mortality (%) and difference in absolute mortality risk (AMR) by socioeconomic indicators, residential area, and age groups

| **Indicator** | **Age**  **Group (years)** | **Reference** | **Comparison** | **Reference Mortality (%)** | **Comparison Mortality (%)** | **AMR^*^**  **(%)** |
| --- | --- | --- | --- | --- | --- | --- |
| Insurance  status | 20–39 | NHI | MA | 22.3 | 40.1 | 17.9 |
|  | 40–64 | NHI | MA | 35.0 | 64.8 | 29.8 |
|  | ≥65 | NHI | MA | 74.0 | 86.7 | 12.7 |
| Income level | 20–39 | Q4 | Q3 | 19.2 | 19.5 | 0.3 |
|  |  |  | Q2 |  | 23.0 | 3.8 |
|  |  |  | Q1 |  | 27.4 | 8.2 |
|  |  |  | MA |  | 40.1 | 20.9 |
|  | 40–64 | Q4 | Q3 | 27.6 | 32.2 | 4.6 |
|  |  |  | Q2 |  | 37.6 | 10.0 |
|  |  |  | Q1 |  | 41.8 | 14.2 |
|  |  |  | MA |  | 64.8 | 37.2 |
|  | ≥65 | Q4 | Q3 | 75.1 | 73.1 | −2.0 |
|  |  |  | Q2 |  | 72.6 | −2.4 |
|  |  |  | Q1 |  | 75.4 | 0.4 |
|  |  |  | MA |  | 86.7 | 11.7 |
| NHIS eligibility status | 20–39 | Employee | Dependent | 13.2 | 23.4 | 10.2 |
|  |  |  | Self-employed (head) |  | 30.8 | 17.5 |
|  |  |  | Self-employed (member) |  | 37.6 | 24.4 |
|  |  |  | MA |  | 40.1 | 26.9 |
|  | 40–64 | Employee | Dependent | 26.7 | 39.2 | 12.5 |
|  |  |  | Self-employed (head) |  | 38.7 | 12.0 |
|  |  |  | Self-employed (member) |  | 40.4 | 13.7 |
|  |  |  | MA |  | 64.8 | 38.1 |
|  | ≥65 | Employee | Dependent | 51.1 | 76.0 | 24.9 |
|  |  |  | Self-employed (head) |  | 71.5 | 20.4 |
|  |  |  | Self-employed (member) |  | 79.3 | 28.2 |
|  |  |  | MA |  | 86.7 | 35.6 |
| Residential area | 20–39 | Seoul metropolitan area | Non-capital metropolitan cities | 24.9 | 22.3 | −2.5 |
|  |  |  | Other regions |  | 22.1 | −2.8 |
|  | 40–64 | Seoul metropolitan area | Non-capital metropolitan cities | 38.1 | 38.2 | 0.1 |
|  |  |  | Other regions |  | 38.5 | 0.4 |
|  | ≥65 | Seoul metropolitan area | Non-capital metropolitan cities | 74.6 | 72.4 | −2.2 |
|  |  |  | Other regions |  | 77.1 | 2.4 |

*AMR was calculated by comparing the mortality and the reference mortality.
