# Supplementary material for: Socioeconomic and Regional Disparities in Long-Term Mortality Among Intensive Care Unit Survivors in South Korea: Nationwide Cohort Study
Source: JMIR Public Health Surveill. 2026 Aug 31;12:e89127. doi: 10.2196/89127 (PMC13529258; doi:10.2196/89127)
Supplement: Supplementary file 4 [file publichealth-v12-e89127-s004.docx]

**Supplementary Table 3**. Adjusted associations between age (years), socioeconomic factors, residential area, and long-term mortality among ICU survivors

|  |  | **1-year** | **2-year** | **4-year** | **6-year** | **8-year** |
| --- | --- | --- | --- | --- | --- | --- |
|  |  | **aHR**  **(95% CI)** | **aHR**  **(95% CI)** | **aHR**  **(95% CI)** | **aHR**  **(95% CI)** | **aHR**  **(95% CI)** |
| Insurance Status | |  |  |  |  |  |
| 20–39 | NHI | Ref | Ref | Ref | Ref | Ref |
|  | MA | 1.12  (0.98–1.28) | 1.21  (1.09–1.34) | 1.35  (1.24–1.46) | 1.45  (1.35–1.57) | 1.48  (1.38–1.60) |
| 40–64 | NHI | Ref | Ref | Ref | Ref | Ref |
|  | MA | 1.28  (1.24–1.32) | 1.37  (1.34–1.41) | 1.51  (1.48–1.54) | 1.60  (1.57–1.63) | 1.63  (1.60–1.66) |
| ≥65 | NHI | Ref | Ref | Ref | Ref | Ref |
|  | MA | 0.97  (0.95–0.98) | 0.99  (0.98–1.01) | 1.02  (1.01–1.04) | 1.04  (1.03–1.05) | 1.05  (1.03–1.06) |
| NHIS eligibility status | |  |  |  |  |  |
| 20–39 | Employee-insured | Ref | Ref | Ref | Ref | Ref |
|  | Dependents | 1.05  (0.91–1.20) | 1.12  (1.00–1.26) | 1.45  (1.31–1.60) | 1.61  (1.46–1.77) | 1.65  (1.50–1.81) |
|  | Self-employed-insured | 1.09  (0.94–1.27) | 1.10  (0.97–1.25) | 1.41  (1.26–1.57) | 1.51  (1.36–1.67) | 1.55  (1.40–1.71) |
|  | Household members | 1.25  (1.09–1.43) | 1.28  (1.14–1.44) | 1.64  (1.48–1.82) | 1.81  (1.65–2.00) | 1.85  (1.68–2.04) |
|  | MA | 1.23  (1.04–1.45) | 1.38  (1.20–1.58) | 1.89  (1.69–2.11) | 2.20  (1.98–2.44) | 2.28  (2.06–2.52) |
| 40–64 | Employee-insured | Ref | Ref | Ref | Ref | Ref |
|  | Dependents | 1.43  (1.37–1.48) | 1.61  (1.56–1.66) | 1.84  (1.79–1.89) | 1.95  (1.90–2.00) | 1.99  (1.94–2.04) |
|  | Self-employed-insured | 1.02  (0.98–1.06) | 1.10  (1.07–1.14) | 1.26  (1.23–1.30) | 1.35  (1.32–1.39) | 1.39  (1.35–1.42) |
|  | Household members | 2.22  (2.13–2.31) | 2.40  (2.32–2.49) | 2.58  (2.50–2.66) | 2.62  (2.55–2.70) | 2.64  (2.56–2.72) |
|  | MA | 1.66  (1.59–1.73) | 1.93  (1.86–1.99) | 2.32  (2.26–2.39) | 2.57  (2.50–2.64) | 2.66  (2.59–2.73) |
| ≥65 | Employee-insured | Ref | Ref | Ref | Ref | Ref |
|  | Dependents | 1.59  (1.53–1.64) | 1.67  (1.62–1.73) | 1.79  (1.74–1.84) | 1.83  (1.78–1.88) | 1.84  (1.80–1.89) |
|  | Self-employed-insured | 1.08  (1.03–1.12) | 1.16  (1.12–1.20) | 1.32  (1.28–1.36) | 1.40  (1.37–1.45) | 1.44  (1.40–1.48) |
|  | Household members | 2.45  (2.36–2.55) | 2.61  (2.52–2.70) | 2.73  (2.64–2.81) | 2.71  (2.64–2.79) | 2.71  (2.63–2.79) |
|  | MA | 1.54  (1.48–1.60) | 1.66  (1.60–1.72) | 1.83  (1.78–1.89) | 1.90  (1.85–1.96) | 1.93  (1.88–1.99) |
| Income Level | |  |  |  |  |  |
| 20–39 | Quartile 4 | Ref | Ref | Ref | Ref | Ref |
|  | Quartile 3 | 1.11  (0.97–1.26) | 1.06  (0.95–1.18) | 1.03  (0.93–1.13) | 1.03  (0.94–1.13) | 1.03  (0.95–1.13) |
|  | Quartile 2 | 1.10  (0.97–1.25) | 1.01  (0.90–1.13) | 1.03  (0.94–1.13) | 1.04  (0.95–1.13) | 1.04  (0.95–1.13) |
|  | Quartile 1 | 1.14  (1.00–1.30) | 1.06  (0.95–1.18) | 1.10  (1.00–1.21) | 1.14  (1.04–1.24) | 1.15  (1.05–1.25) |
|  | MA | 1.22  (1.04–1.42) | 1.25  (1.10–1.42) | 1.40  (1.26–1.55) | 1.53  (1.39–1.69) | 1.57  (1.43–1.72) |
| 40–64 | Quartile 4 | Ref | Ref | Ref | Ref | Ref |
|  | Quartile 3 | 1.19  (1.15–1.23) | 1.17  (1.14–1.21) | 1.19  (1.16–1.22) | 1.20  (1.17–1.22) | 1.20  (1.17–1.23) |
|  | Quartile 2 | 1.34  (1.29–1.39) | 1.32  (1.28–1.36) | 1.32  (1.28–1.35) | 1.32  (1.29–1.35) | 1.32  (1.29–1.35) |
|  | Quartile 1 | 1.35  (1.30–1.40) | 1.31  (1.27–1.35) | 1.29  (1.26–1.32) | 1.30  (1.27–1.33) | 1.30  (1.27–1.33) |
|  | MA | 1.56  (1.49–1.62) | 1.64  (1.59–1.70) | 1.80  (1.75–1.84) | 1.91  (1.87–1.96) | 1.95  (1.91–2.00) |
| ≥65 | Quartile 4 | Ref | Ref | Ref | Ref | Ref |
|  | Quartile 3 | 1.11  (1.09–1.13) | 1.11  (1.09–1.12) | 1.09  (1.07–1.10) | 1.09  (1.07–1.10) | 1.08  (1.07–1.10) |
|  | Quartile 2 | 1.15  (1.13–1.17) | 1.14  (1.12–1.16) | 1.13  (1.11–1.14) | 1.13  (1.11–1.14) | 1.13  (1.11–1.14) |
|  | Quartile 1 | 1.17  (1.15–1.19) | 1.17  (1.15–1.18) | 1.16  (1.14–1.17) | 1.15  (1.14–1.17) | 1.16  (1.14–1.17) |
|  | MA | 1.04  (1.02–1.06) | 1.06  (1.04–1.08) | 1.09  (1.07–1.11) | 1.11  (1.09–1.12) | 1.11  (1.10–1.13) |
| Residential area | |  |  |  |  |  |
| 20–39 | Seoul metropolitan area | Ref | Ref | Ref | Ref | Ref |
|  | Non-capital metropolitan cities | 1.05  (0.94–1.18) | 1.01  (0.92–1.11) | 1.00  (0.93–1.09) | 1.02  (0.95–1.10) | 1.02  (0.95–1.10) |
|  | Other regions | 1.15  (1.05–1.27) | 1.11  (1.02–1.20) | 1.10  (1.03–1.18) | 1.11  (1.04–1.18) | 1.11  (1.04–1.18) |
| 40–64 | Seoul metropolitan area | Ref | Ref | Ref | Ref | Ref |
|  | Non-capital metropolitan cities | 0.96  (0.93–1.00) | 0.96  (0.93–0.99) | 0.95  (0.93–0.97) | 0.95  (0.93–0.97) | 0.96  (0.94–0.98) |
|  | Other regions | 1.11  (1.08–1.14) | 1.09  (1.07–1.11) | 1.05  (1.04–1.07) | 1.06  (1.04–1.07) | 1.06  (1.04–1.07) |
| ≥65 | Seoul metropolitan area | Ref | Ref | Ref | Ref | Ref |
|  | Non-capital metropolitan cities | 0.99  (0.97–1.01) | 0.99  (0.98–1.01) | 1.10  (0.99–1.02) | 1.01  (1.00–1.03) | 1.01  (1.00–1.03) |
|  | Other regions | 1.15  (1.13–1.16) | 1.13  (1.11–1.14) | 1.11  (1.10–1.12) | 1.11  (1.10–1.12) | 1.11  (1.10–1.12) |

All models were adjusted for age as a continuous variable, sex, smoking status, Charlson Comorbidity Index score, and interventions. CI, confidence interval; aHR, adjusted hazard ratio; MA, Medical Aid; NHI, National Health Insurance; Ref, Reference.
