# Supplementary material for: Socioeconomic and Regional Disparities in Long-Term Mortality Among Intensive Care Unit Survivors in South Korea: Nationwide Cohort Study
Source: JMIR Public Health Surveill. 2026 Aug 31;12:e89127. doi: 10.2196/89127 (PMC13529258; doi:10.2196/89127)
Supplement: Supplementary file 5 [file publichealth-v12-e89127-s005.docx]

**Supplementary Table 4**. Age group-specific associations between residential area and long-term mortality among ICU survivors after adjustment for individual socioeconomic indicators.

|  |  | **1-year** | **2-year** | **4-year** | **6-year** | **8-year** |
| --- | --- | --- | --- | --- | --- | --- |
|  |  | **aHR**  **(95% CI)** | **aHR**  **(95% CI)** | **aHR**  **(95% CI)** | **aHR**  **(95% CI)** | **aHR**  **(95% CI)** |
| Adjusted insurance status | |  |  |  |  |  |
| 20–39 | Seoul metropolitan area | Ref | Ref | Ref | Ref | Ref |
|  | Non-capital metropolitan cities | 1.07  (0.94–1.23) | 1.05  (0.94–1.19) | 1.03  (0.93–1.14) | 1.04  (0.94–1.14) | 1.02  (0.93–1.12) |
|  | Other regions | 1.27  (1.13–1.42) | 1.21  (1.09–1.33) | 1.15  (1.06–1.25) | 1.15  (1.06–1.25) | 1.14  (1.05–1.23) |
| 40–64 | Seoul metropolitan area | Ref | Ref | Ref | Ref | Ref |
|  | Non-capital metropolitan cities | 0.97  (0.93–1.01) | 0.94  (0.91–0.98) | 0.92  (0.90–0.95) | 0.92  (0.90–0.95) | 0.93  (0.90–0.95) |
|  | Other regions | 1.13  (1.09–1.16) | 1.11  (1.08–1.14) | 1.07  (1.04–1.09) | 1.06  (1.04–1.08) | 1.06  (1.04–1.08) |
| ≥65 | Seoul metropolitan area | Ref | Ref | Ref | Ref | Ref |
|  | Non-capital metropolitan cities | 1.01  (0.99–1.03) | 1.00  (0.98–1.02) | 1.01  (0.99–1.02) | 1.01  (1.00–1.03) | 1.01  (1.00–1.03) |
|  | Other regions | 1.17  (1.15–1.19) | 1.15  (1.14–1.17) | 1.13  (1.12–1.15) | 1.13  (1.12–1.14) | 1.13  (1.11–1.14) |
| Adjusted NHIS eligibility status | |  |  |  |  |  |
| 20–39 | Seoul metropolitan area | Ref | Ref | Ref | Ref | Ref |
|  | Non-capital metropolitan cities | 1.07  (0.93–1.23) | 1.05  (0.93–1.18) | 1.02  (0.92–1.13) | 1.02  (0.93–1.13) | 1.01  (0.91–1.11) |
|  | Other regions | 1.26  (1.12–1.41) | 1.20  (1.09–1.32) | 1.14  (1.04–1.24) | 1.14  (1.05–1.23) | 1.12  (1.04–1.22) |
| 40–64 | Seoul metropolitan area | Ref | Ref | Ref | Ref | Ref |
|  | Non-capital metropolitan cities | 0.96  (0.93–1.01) | 0.94  (0.91–0.97) | 0.92  (0.89–0.95) | 0.92  (0.89–0.94) | 0.92  (0.89–0.94) |
|  | Other regions | 1.11  (1.08–1.15) | 1.09  (1.06–1.12) | 1.05  (1.03–1.08) | 1.04  (1.02–1.06) | 1.04  (1.02–1.06) |
| ≥65 | Seoul metropolitan area | Ref | Ref | Ref | Ref | Ref |
|  | Non-capital metropolitan cities | 1.01  (0.99–1.03) | 1.00  (0.98–1.02) | 1.01  (0.99–1.03) | 1.01  (1.00–1.03) | 1.01  (1.00–1.03) |
|  | Other regions | 1.18  (1.16–1.20) | 1.16  (1.14–1.17) | 1.14  (1.12–1.15) | 1.13  (1.12–1.15) | 1.13  (1.12–1.14) |
| Adjusted income level | |  |  |  |  |  |
| 20–39 | Seoul metropolitan area | Ref | Ref | Ref | Ref | Ref |
|  | Non-capital metropolitan cities | 1.07  (0.93–1.23) | 1.05  (0.93–1.19) | 1.03  (0.92–1.14) | 1.03  (0.94–1.14) | 1.02  (0.92–1.12) |
|  | Other regions | 1.26  (1.13–1.41) | 1.20  (1.09–1.33) | 1.15  (1.05–1.25) | 1.14  (1.06–1.24) | 1.13  (1.05–1.23) |
| 40–64 | Seoul metropolitan area | Ref | Ref | Ref | Ref | Ref |
|  | Non-capital metropolitan cities | 0.97  (0.93–1.01) | 0.94  (0.91–0.98) | 0.92  (0.90–0.95) | 0.93  (0.90–0.95) | 0.93  (0.90–0.95) |
|  | Other regions | 1.12  (1.09–1.16) | 1.11  (1.08–1.14) | 1.07  (1.04–1.09) | 1.06  (1.03–1.08) | 1.05  (1.03–1.08) |
| ≥65 | Seoul metropolitan area | Ref | Ref | Ref | Ref | Ref |
|  | Non-capital metropolitan cities | 1.01  (0.99–1.04) | 1.01  (0.99–1.03) | 1.01  (0.99–1.03) | 1.01  (1.00–1.03) | 1.01  (1.00–1.03) |
|  | Other regions | 1.17  (1.15–1.19) | 1.15  (1.13–1.17) | 1.13  (1.12–1.14) | 1.13  (1.12–1.14) | 1.13  (1.11–1.14) |

All models were adjusted for age as a continuous variable, sex, smoking status, Charlson Comorbidity Index score, interventions, and the corresponding socioeconomic indicator. Separate models were fitted for each socioeconomic indicator: insurance status, NHIS eligibility status, and income level. CI, confidence interval; aHR, adjusted hazard ratio; Ref, Reference.
