# Supplementary material for: Socioeconomic and Regional Disparities in Long-Term Mortality Among Intensive Care Unit Survivors in South Korea: Nationwide Cohort Study
Source: JMIR Public Health Surveill. 2026 Aug 31;12:e89127. doi: 10.2196/89127 (PMC13529258; doi:10.2196/89127)
Supplement: Supplementary file 6 [file publichealth-v12-e89127-s006.docx]

**Supplementary Table 5**. Comparison of characteristics between survivors and decedents in the low-income group (MA and 1st quartile)

|  | **Total** | **Survivors** | **Decedents** | ***p*-value** |
| --- | --- | --- | --- | --- |
| Number of patients, n (%) | 218,559 (100.0) | 115,369 (52.8) | 103,190 (47.2) |  |
| Age, mean ± SD | 65.9±14.8 | 60.9±13.5 | 71.4±14.2 | <0.001 |
| Sex |  |  |  | <0.001 |
| Male | 120,931 (55.3) | 64,669 (56.1) | 56,262 (54.5) |  |
| Female | 97,628 (44.7) | 50,700 (44.0) | 46,928 (45.5) |  |
| Income |  |  |  | <0.001 |
| MA | 83,766 (38.3) | 35,716 (31.0) | 48,050 (46.6) |  |
| Quartile 1 (Lowest) | 134,793 (61.7) | 79,653 (69.0) | 55,140 (53.4) |  |
| Residential area |  |  |  | <0.001 |
| Seoul metropolitan area | 97,089 (44.4) | 51,902 (45.0) | 45,187 (43.8) |  |
| Non-capital metropolitan cities | 37,897 (17.3) | 21,607 (18.7) | 16,290 (15.8) |  |
| Other regions | 83,573 (38.2) | 41,860 (36.3) | 41,713 (40.4) |  |
| Smoking status |  |  |  | <0.001 |
| Non-smoker | 65,755 (30.1) | 43,459 (37.7) | 22,296 (21.6) |  |
| Ex-smoker | 27,827 (12.7) | 19,568 (17.0) | 8,259 (8.0) |  |
| Current smoker | 25,504 (11.7) | 16,369 (14.2) | 9,135 (8.9) |  |
| Unknown | 99,473 (45.5) | 35,973 (31.2) | 63,500 (61.5) |  |
| Comorbidities |  |  |  |  |
| CCI score, mean ± SD | 3.3±2.6 | 2.7±2.3 | 3.9±2.7 | <0.001 |
| Hypertension | 138,017 (63.2) | 65,877 (57.1) | 72,140 (69.9) | <0.001 |
| Dyslipidemia | 135,952 (62.2) | 70,616 (61.2) | 65,336 (63.3) | <0.001 |
| CAD | 11,228 (5.1) | 4,988 (4.3) | 6,240 (6.1) | <0.001 |
| Diabetes | 101,972 (46.7) | 47,674 (41.3) | 54,298 (52.6) | <0.001 |
| Stroke | 32,629 (14.9) | 12,576 (10.9) | 20,053 (19.4) | <0.001 |
| Acute myocardial infarction | 9,592 (4.4) | 4,284 (3.7) | 5,308 (5.1) | <0.001 |
| Congestive heart failure | 32,567 (14.9) | 13,067 (11.3) | 19,500 (18.9) | <0.001 |
| Peripheral vascular disease | 46,035 (21.1) | 22,672 (19.7) | 23,363 (22.6) | <0.001 |
| Cerebral vascular accident | 59,423 (27.2) | 26,028 (22.6) | 33,395 (32.4) | <0.001 |
| Dementia | 29,508 (13.5) | 6,712 (5.8) | 22,796 (22.1) | <0.001 |
| Pulmonary disease | 107,245 (49.1) | 51,511 (44.7) | 55,734 (54.0) | <0.001 |
| Connective tissue disorder | 12,840 (5.9) | 6,633 (5.8) | 6,207 (6.0) | 0.008 |
| Peptic ulcer | 86,425 (39.5) | 44,258 (38.4) | 42,167 (40.9) | <0.001 |
| Liver disease | 22,857 (10.5) | 9,761 (8.5) | 13,096 (12.7) | <0.001 |
| Diabetes complications | 32,964 (15.1) | 13,888 (12.0) | 19,076 (18.5) | <0.001 |
| Paraplegia | 6,285 (2.9) | 2,197 (1.9) | 4,088 (4.0) | <0.001 |
| Renal disease | 22,889 (10.5) | 8,364 (7.3) | 14,525 (14.1) | <0.001 |
| Cancer | 33,689 (15.4) | 14,214 (12.3) | 19,475 (18.9) | <0.001 |
| Metastatic cancer | 5,458 (2.5) | 1,321 (1.2) | 4,137 (4.0) | <0.001 |
| Severe liver disease | 3,464 (1.6) | 1,034 (0.9) | 2,430 (2.4) | <0.001 |
| HIV | 169 (0.1) | 101 (0.1) | 68 (0.1) | 0.069 |
| Intervention |  |  |  |  |
| Mechanical ventilation | 39,590 (18.1) | 18,247 (15.8) | 21,343 (20.7) | <0.001 |
| Renal replacement | 6,724 (3.1) | 2,364 (2.1) | 4,360 (4.2) | <0.001 |
| Vasopressor or inotropic drugs | 89,615 (41.0) | 46,964 (40.7) | 42,651 (41.3) | 0.003 |
| ECMO | 748 (0.3) | 431 (0.4) | 317 (0.3) | 0.008 |
| Hospitalization Type |  |  |  | <0.001 |
| Tertiary hospital^*^ | 74,975 (34.3) | 44,578 (38.6) | 30,397 (29.5) |  |
| General hospital | 143,584 (65.7) | 70,791 (61.4) | 72,793 (70.5) |  |

*Ministry–designated tertiary hospitals in Korea. CAD, coronary artery disease; CCI, Charlson Comorbidity Index; ECMO, extracorporeal membrane oxygenation; HIV, human immunodeficiency virus; MA, Medical Aid; SD, standard deviation.
