# Supplementary material for: Socioeconomic and Regional Disparities in Long-Term Mortality Among Intensive Care Unit Survivors in South Korea: Nationwide Cohort Study
Source: JMIR Public Health Surveill. 2026 Aug 31;12:e89127. doi: 10.2196/89127 (PMC13529258; doi:10.2196/89127)
Supplement: Supplementary file 7 [file publichealth-v12-e89127-s001.docx]

**Supplementary Figure 1**. Study design and assessment windows
